# The association between the serum fat-soluble vitamins (A, D and E) and the intake of live microbes: a national population based cross-sectional study

**DOI:** 10.3389/fnut.2025.1593461

**Published:** 2025-07-29

**Authors:** Huiling Zheng, Chan Liu, Dan Xu, Mei Li, Hua Hong

**Affiliations:** ^1^Department of Health Management Centre, The First Affiliated Hospital of Sun Yat-sen University, Guangzhou, China; ^2^Department of Nephrology, The First Affiliated Hospital of Sun Yat-sen University, Key Laboratory of Nephrology, Ministry of Health of China, Guangdong Provincial Key Laboratory of Nephrology, Guangzhou, China; ^3^The First Affiliated Hospital, Sun Yat-Sen University, Guangzhou, China; ^4^CCRE, Curtin School of Population Health, Faculty of Health Sciences, Curtin University, Bentley, WA, Australia; ^5^Curtin Medical School, Faculty of Health Sciences, Curtin University, Bentley, WA, Australia; ^6^Department of Gastroenterology, The First Affiliated Hospital of Sun Yat-sen University, Guangzhou, China

**Keywords:** fat-soluble vitamins, live microbes, NHANES, cross-sectional analysis, the exposure-response curves

## Abstract

**Objective:**

Fat-soluble vitamins (FSVs) play essential roles in numerous physiological processes and are involved in the onset and progression of chronic diseases. However, limited research has investigated whether dietary intake of live microbes correlates with circulating FSVs levels. This study aims to explore the relationship between the dietary intake of live microbes and the serum levels of FSVs.

**Methods:**

We conducted a cross-sectional analysis on a nationally representative sample of 27,668 participants from the National Health and Nutrition Examination Survey (NHANES) to assess the association between serum levels of FSVs and the intake of dietary live microbes. Weighted generalized linear regression and logistic regression models were used to evaluate the associations, adjusting for demographic, lifestyle, laboratory, and dietary covariates.

**Results:**

After multivariate adjustment, each one-unit increase in the natural log-transformed MedHi food intake corresponds to an increase of 0.17 μg/dL in vitamin A (95% CI: 0.04, 0.30), 0.36 nmol/L in vitamin D (95% CI: 0.22, 0.51), and 4.65 μg/dL in vitamin E (95% CI: 1.91, 7.39). Furthermore, the exposure-response curves for MedHi consumption showed a consistent decreasing trend in the prevalence of low serum levels of these FSVs.

**Conclusion:**

In conclusion, this study provides evidence that the dietary intake of live microbes is associated with increased serum levels of FSVs and may contribute to reducing deficiencies in these vitamins.

## Introduction

FSVs are essential micronutrients involved in numerous physiological and biochemical processes such as vision, bone metabolism, immune regulation, and antioxidant defense (1–4). Deficiency or imbalance of FSVs has been associated with various chronic diseases, including osteoporosis, cardiovascular disease, and metabolic disorders (5–7). Emerging evidence suggests that the gut microbiota may modulate the absorption, metabolism, and bioavailability of FSVs (8). The gut microbiota can influence bile acid metabolism, lipid absorption pathways, and vitamin receptor expression, all of which are closely related to FSVs transport and utilization. However, while previous studies have explored the interaction between gut microbiota and vitamin metabolism, few have directly examined whether the dietary intake of live microbes influences serum FSVs levels in large, population-based samples (9). This study aims to address this knowledge gap.

The composition of the gut microbiota is influenced by endogenous and environmental factors, including diet, antibiotic intake, and exogenous substances. Among these factors, diet is regarded as the main driver of changes in gut bacterial diversity, potentially influencing its functional relationship with the host (10). Recent studies have shown that certain foodborne microbes can survive gastrointestinal transit and potentially colonize or interact with the host gut microbiome, thus affecting metabolic processes (11). Although studies have confirmed that the gut microbiota and its metabolites facilitate the absorption of FSVs (8, 12, 13), limited research has evaluated whether the dietary intake level of live microbes is associated with serum FSVs concentrations in population-based settings.

Some studies have also investigated the interaction between vitamin D and gut microbiota. For instance, vitamin D supplementation has been shown to alter the composition of gut microbiota by increasing the abundance of Firmicutes, Actinobacteria, and Bacteroidetes (14, 15), while population studies have reported that individuals with higher vitamin D levels tend to have greater gut microbial diversity (7, 16). These findings suggest potential bidirectional interactions between FSVs and gut microbiota. However, these studies primarily focused on vitamin D, while less is known about how live microbial intake may influence overall FSVs status.

Given that previous evidence is mostly limited to mechanistic and intervention studies, and that data from large-scale population studies are lacking, we aimed to investigate the association between dietary intake of live microbes and serum levels of FSVs using a nationally representative sample from NHANES. Cross-sectional surveys are flexible in design, allowing biomarkers to be measured in a short timeframe during the survey as needed for scientific purposes (17). This cross-sectional analysis may provide new insights into the potential role of the dietary intake of live microbes in modulating FSVs levels.

## Methods

### Study population

This study was a secondary analysis based on publicly available, de-identified data from the National Health and Nutrition Examination Survey (NHANES), a cross-sectional, nationally representative survey conducted in the United States. All data collection procedures, including physical examinations, biomarker assessments, and laboratory testing, were conducted by trained NHANES staff following standardized protocols. Informed consent was obtained from all participants by NHANES at the time of data collection. The authors did not perform any additional data collection. All NHANES data are publicly accessible through the CDC website.

These surveys gather comprehensive data on nutritional intake, health behaviors, and medical conditions. Trained interviewers performed in-home interviews, collecting detailed demographic, dietary, socioeconomic, and health-related information using standardized questionnaires. Physical examinations were conducted at a mobile examination center.

**Figure 1:**
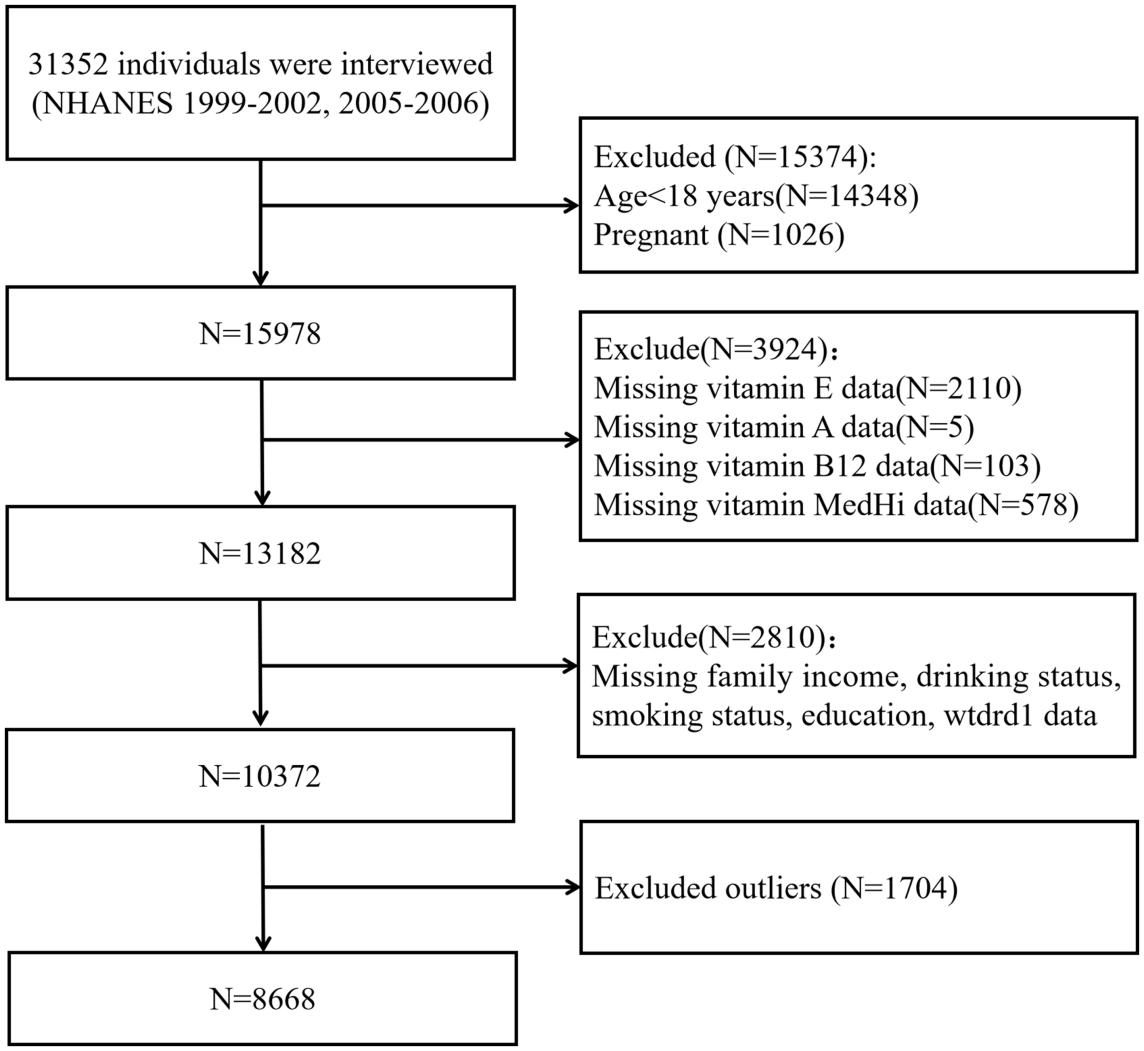
Flowchart of participant selection for Group 1 (NHANES 1999–2002 and 2005–2006).

**Figure 2:**
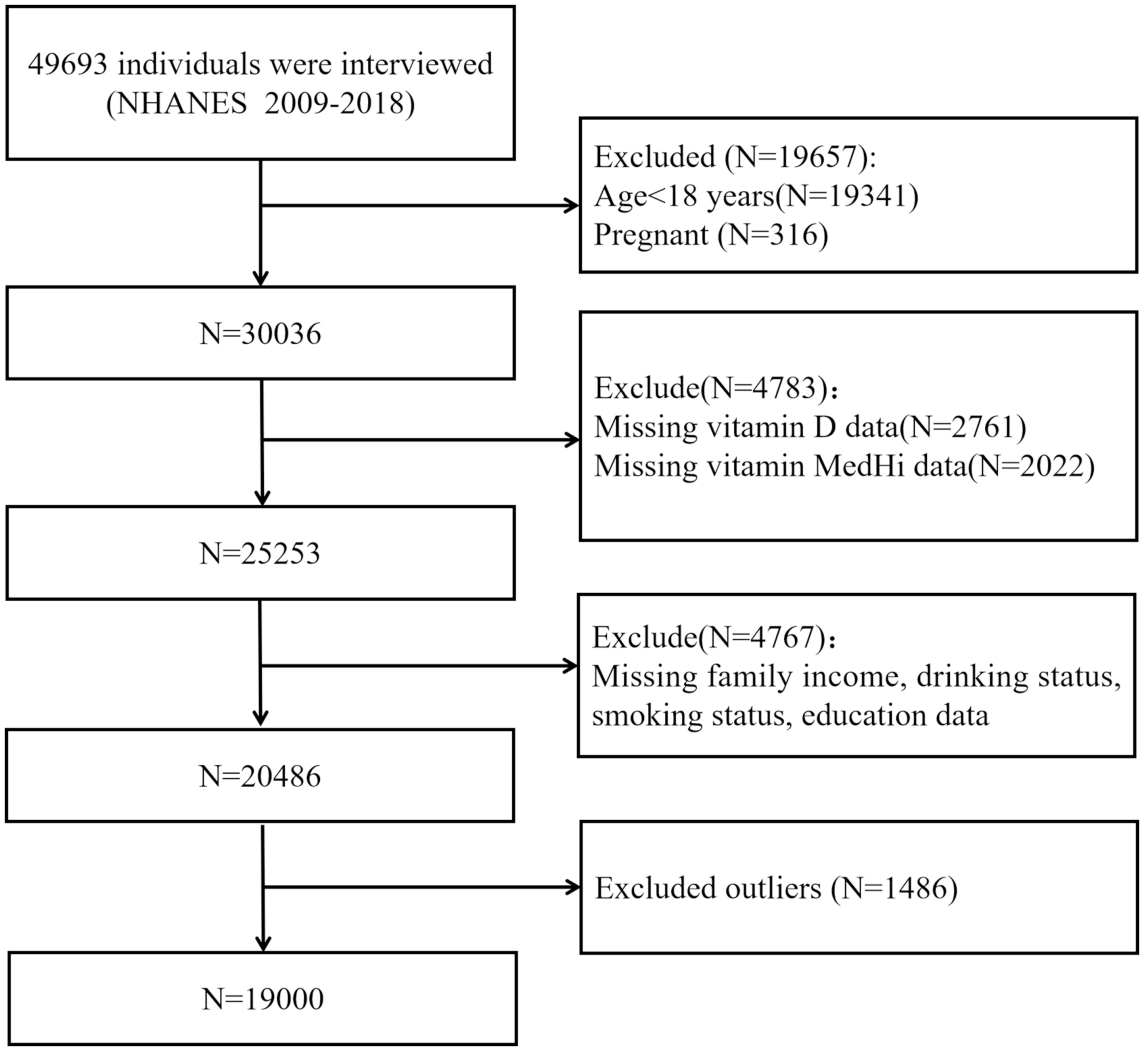
Flowchart of participant selection for Group 2 (NHANES 2009–2018).

We restricted our study sample to individuals over the age of 18 and excluded pregnant individuals or those with missing data on missing data on exposures, covariates, or outcomes. Due to differences in biomarker availability across survey cycles, participants were divided into two groups: Group 1 (1999–2002 and 2005–2006) for analysis of vitamins A, E, and B12; Group 2 (2009–2018) for analysis of vitamin D. This grouping ensured appropriate use of available data while maximizing the analytical sample size. The inclusion and exclusion criteria for these groups are illustrated in Figure 1 for Group 1, and in Figure 2 for Group 2. All participants provided written informed consent, and the National Center for Health Statistics (NCHS) Research Ethics Review Board approved the survey protocol. As this study constituted a secondary analysis of de-identified data, it did not require additional institutional review board approval.

### Dietary intake of live microbes category

The dietary data are collected using an in-person 24-h dietary recall component. NCHS collaborated with the US Department of Agriculture (USDA) to compare the 24-h dietary data against the USDA Food and Nutrient Database, estimating nutrient and energy intakes. Subsequently, sanders devised a method to quantify the live microbial content (per gram) in 9,388 food codes across 48 subgroups within the NHANES database (14). Four experts in the field (MLM, MES, RH, and CH) categorized the live microbial content of foods into three levels: low (Lo; <10^4 CFU/g), medium (Med; 10^4–10^7 CFU/g), and high (Hi; >10^7 CFU/g). Additionally, a composite category ‘MedHi’ was established by including consumers’ intake of foods from the ‘Med’, ‘Hi’, or both categories. Consensus was required among the panel of experts with disagreements resolved by panel discussion.

In this study, individuals were classified using two distinct methods based on their consumption of dietary live microbes. Participants were divided into three groups according to their MedHi consumption levels to assess live microbe ingestion: G1 (those without any MedHi food intake), G2 (those consuming MedHi foods above zero but below the median consumption level), and G3 (those consuming MedHi foods above the median level). Additionally, participants were divided into three groups based on the general microbial content of their diets: low (consumed only Lo-category foods), moderate (consumed Med-category foods but no Hi-category foods), and high (consumed any Hi-category foods).

### Vitamins

Serum vitamin concentrations were extracted from the dataset and included as exposure variables. A standardized and fully validated technique employing liquid chromatography–tandem mass spectrometry (LC–MS/MS) was utilized for the quantitative assessment of 25-hydroxyvitamin D3 (25OHD3), 3-epi-25-hydroxyvitamin D3 (epi-25OHD3), and 25-hydroxyvitamin D2 (25OHD2) in the serum of all eligible participants. Total serum 25(OH)D was defined as the combined concentrations of 25(OH)D3 and 25(OH)D2. The quality assurance and quality control protocols of the NHANES comply with the mandates of the 1988 Clinical Laboratory Improvement Act. In addition to FSVs, vitamin B12 (a water-soluble vitamin) was included for comparison to assess whether associations with dietary live microbes were specific to FSVs. Vitamins were categorized into two groups: FSVs, represented by vitamins A, D, and E, and water-soluble vitamins, represented by vitamin B12. Deficiency in vitamin D was defined as levels below 50 nmol/L (18–20) for individuals classified as the low concentration group. Concentrations of vitamins A, E, and B12 were ranked from lowest to highest and divided at the median into low and high concentration groups. All vitamins were analyzed both as continuous and categorical variables.

### Covariates

Potential confounders were evaluated as covariates. Questionnaires collected information on age, sex, race (Non-Hispanic White, Non-Hispanic Black, Mexican-American, Other Hispanic, Other Race), educational level (less than high school, high school or equivalent, college or above), family income-to-poverty ratio (PIR)(<1.3, 1.3–3.5, >3.5), smoking status (never smoker, former smoker, current smoker), and leisure-time physical activity (inactive, moderately active, vigorously active). Drinking status was categorized into four groups: never (fewer than 12 drinks in a lifetime), former (no drinking in the past year but ≥12 drinks previously), light (up to 1 drink per day for women or 2 drinks per day for men in the past year), and heavy (more than 1 drink per day for women or 2 drinks per day for men on average over the past year). Laboratory analysis covariates included serum total cholesterol (TC), serum alanine aminotransferase (ALT). Obesity was defined according to WHO standards as a BMI ≥ 30.0 kg/m^2^. Individuals were classified as hypertensive based on one or more of the following criteria: a self-reported doctor’s diagnosis of hypertension, current use of antihypertensive medications, a systolic blood pressure of 140 mmHg or higher, or a diastolic blood pressure of 90 mmHg or higher. Diabetes was defined based on any of the following criteria: a 2-h plasma glucose level ≥ 11.1 mmol/L, an HbA1c level ≥ 6.5%, a fasting plasma glucose level ≥ 7.0 mmol/L, current use of diabetic medications or insulin, or a self-reported physician diagnosis. Daily dietary variables assessed included dietary vitamin A, D, E, B12 intake, total energy. Chronic kidney disease (CKD) was defined as estimated glomerular filtration rate < 60 mL/min/1.73 m^2^ and/or urinary albumin to creatinine ratio >30 mg/g (21). Time spent outdoors during a typical workday and non-work day were measured with two questions: “The next questions ask about the time you spent outdoors during the past 30 days. By outdoors, I mean outside and not under any shade. How much time did you usually spend outdoors between 9 in the morning and 5 in the afternoon on the days that you worked or went to school” and “During the past 30 days, how much time did you usually spend outdoors between 9 in the morning and 5 in the afternoon on the days when you were not working or going to school?” Total time spent outdoors was grouped into time intervals: 30 min or less, 30 min to 1 h, 1–2 h, 2–3 h, 3–4 h, 4–5 h, 5–6 h, 6–8 h, 8–10 h, 10–12 h, 12–14 h, 14–16 h.

### Statistical analyses

One-day dietary weights were applied in the analyses. Continuous variables are presented as the mean (standard deviation) or median (interquartile range), while categorical variables are presented as number (percentages).

To facilitate the analysis, the dietary intake of MedHi underwent natural log-transformation after adding 0.1g and was then analyzed as a continuous variable. These regression models adjusted the following covariates: Model 1 was crude model (unadjusted); Model 2 was adjusted for age, sex; Model 3 was adjusted for model 2 plus race, education, smoking status, drinking status, family income and physical activity; Model 4 was adjusted for model 3 plus TC, ALT, chronic disease status (diabetes, hypertension, CKD, obesity), energy (kcal/d) and dietary vitamins intake. For regression models that measure grams MedHi consumed as a continuous exposure, regression coefficients are reported one-unit increment in natural log-transformation. These coefficients indicate the adjusted mean difference in the outcome for one-unit increment in natural log-transformation in exposure. In regression models using the three-level classification of patients based on dietary intakes, non-consumers (G1) serve as the reference group. Regression coefficients are reported for the two indicator variables: one for participants with intakes above zero but below the median (G2), and another for participants with intakes at or above the median (G3), both included simultaneously in the model.

## Results

After applying inclusion and exclusion criteria, data were divided into two groups: Group 1 comprised 8,668 individuals from the NHANES cycles 1999–2002 and 2005–2006 for examining the association between serum vitamin levels and the intake of live microbes (Figure 1). Group 2 included 19,000 individuals surveyed between 2009 and 2018 for investigating the association between serum vitamin D levels and live microbe intake (Figure 2). Table 1 shows the demographic characteristics of Group 1 (NHANES 1999–2002 and 2005–2006). In Group 1, the mean age was 45.6 years (±0.34), 50.5% were male, and 72.8% were non-Hispanic White. Group 2 displayed comparable demographic distributions (see Supplementary Table S1).

**Table 1:** Demographic characteristics of group 1 (NHANES 1999–2002 and 2005–2006).

Variables	Total	MedHi groups			*p*
		G1	G2	G3	
Age (years)	45.61 (0.34)	44.19 (0.31)	47.30 (0.46)	44.23 (0.66)	<0.0001
Sex					<0.001
Male	4,542 (50.49)	1711 (53.93)	2094 (49.23)	737 (47.54)	
Female	4,126 (49.51)	1,388 (46.07)	1945 (50.77)	793 (52.46)	
Race					<0.0001
Non-Hispanic White	4,345 (72.77)	1,381 (68.38)	2043 (72.90)	921 (79.84)	
Other Race	4,323 (27.23)	1718 (31.62)	1996 (27.10)	609 (20.16)	
Education					<0.0001
Less than high school	1,226 (6.24)	490 (7.45)	575 (6.18)	161 (4.34)	
High school or equivalent	3,551 (39.47)	1,453 (47.34)	1,604 (38.21)	494 (29.06)	
College or above	3,891 (54.29)	1,156 (45.21)	1860 (55.61)	875 (66.60)	
Family income-to-poverty ratio					<0.0001
<1.3	2,358 (20.56)	1,017 (26.91)	1,028 (18.40)	313 (14.72)	
1.3–3.5	3,414 (36.49)	1,265 (40.14)	1,612 (36.58)	537 (30.15)	
>3.5	2,896 (42.95)	817 (32.95)	1,399 (45.02)	680 (55.13)	
Smoking status					<0.0001
Never	4,359 (49.77)	1,460 (45.11)	2087 (51.50)	812 (53.74)	
Former	2,230 (23.99)	700 (20.84)	1,107 (25.51)	423 (25.89)	
Current	2079 (26.25)	939 (34.06)	845 (22.99)	295 (20.37)	
Drinking status					0.004
Non-alcohol intake	1,179 (12.07)	435 (12.05)	572 (12.98)	172 (10.10)	
Former alcohol intake	1734 (16.94)	680 (19.44)	778 (15.71)	276 (15.51)	
Mild alcohol intake	2,826 (34.45)	898 (30.57)	1,383 (35.89)	545 (37.79)	
Heavy alcohol intake	2,929 (36.53)	1,086 (37.95)	1,306 (35.43)	537 (36.60)	
Recreational activity					<0.0001
Inactive	3,352 (32.06)	1,322 (36.65)	1,519 (30.72)	511 (27.47)	
Moderate	193 (1.60)	90 (2.32)	92 (1.51)	11 (0.60)	
Vigorous	5,118 (66.27)	1,685 (61.04)	2,427 (67.77)	1,006 (71.93)	
Obesity					0.57
No	5,642 (66.65)	1969 (66.49)	2,633 (67.87)	1,040 (68.45)	
Yes	2,879 (32.06)	1,064 (33.51)	1,343 (32.13)	472 (31.55)	
CKD					<0.001
No	7,128 (86.51)	2,491 (86.10)	3,314 (86.53)	1,323 (90.13)	
Yes	1,479 (12.79)	581 (13.90)	699 (13.47)	199 (9.87)	
Diabetes					0.17
No	7,521 (90.79)	2,681 (90.74)	3,475 (90.20)	1,365 (92.19)	
Yes	1,147 (9.21)	418 (9.26)	564 (9.80)	165 (7.81)	
Hypertension					0.04
No	5,222 (65.06)	1840 (64.26)	2,390 (64.00)	992 (68.81)	
Yes	3,441 (34.92)	1,257 (35.74)	1,647 (36.00)	537 (31.19)	
TC (mmol/L)	5.15 (0.02)	5.15 (0.03)	5.17 (0.02)	5.12 (0.03)	0.47
ALT (U/L)	25.83 (0.30)	26.36 (0.54)	25.48 (0.37)	25.73 (0.74)	0.41
MedHi (g/d)	88.19 (2.17)	0.00 (0.00)	120.61 (2.32)	164.13 (3.60)	<0.0001
Serum Vitamin A (ug/dL)	59.30 (0.34)	57.94 (0.47)	59.75 (0.44)	60.60 (0.54)	<0.0001
Serum vitamin E (ug/dL)	1183.08 (7.84)	1131.66 (10.66)	1211.08 (9.87)	1207.13 (15.08)	<0.0001
Serum vitamin B12 (pg/mL)	469.75 (2.97)	468.39 (4.64)	473.45 (4.51)	463.78 (4.64)	0.29
Vitamin A intake (mcg)	604.96 (12.49)	538.52 (24.77)	621.00 (15.56)	680.80 (16.80)	<0.001
Vitamin E intake (mg)	7.02 (0.10)	6.03 (0.11)	7.41 (0.17)	7.83 (0.19)	<0.0001
Vitamin B12 intake (mcg)	5.31 (0.11)	5.10 (0.22)	5.35 (0.14)	5.56 (0.17)	0.21
Energy (kcal/d)	2200.67 (18.02)	2098.31 (24.25)	2204.51 (24.16)	2363.90 (29.38)	<0.0001

Data are presented as means (SE) for continuous measures and numbers (percentage) for categorical measures.

### Generalized linear regression analyses

Table 2 presents the results from univariable and multivariable weighted generalized linear regression analyses exploring the correlation between dietary intake of MedHi food and serum vitamin levels. The multivariate adjustment indicated each one-unit increase in the natural log-transformed MedHi food intake corresponding to an increase of 0.17 μg/dL in vitamin A (95% CI: 0.04, 0.30), 0.36 nmol/L in vitamin D (95% CI: 0.22, 0.51), and 4.65 μg/dL in vitamin E (95% CI: 1.91, 7.39). No significant association was found with vitamin B12 (Supplementary Table S2).

**Table 2:** Regression analysis showing the association between dietary intake of live microbes with serum vitamins.

Variables	Per one-unit increment in natural log-transformed MedHi(g)							
	Model 1	*p*	Model 2	*p*	Model 3	*p*	Model 4	*p*
Serum vitamin A (ug/dL)	0.34 [0.21, 0.48]	<0.001	0.33 [0.20, 0.46]	<0.001	0.17 [0.04, 0.30]	0.011	0.17 [0.04, 0.30]	0.011
Serum vitamin D (nmol/L)	1.04 [0.87, 1.21]	<0.001	0.88 [0.71, 1.06]	<0.001	0.45 [0.29, 0.60]	<0.001	0.36 [0.22, 0.51]	<0.001
Serum vitamin E (ug/dL)	13.30[10.19, 16.40]	<0.001	9.86 [6.71, 13.01]	<0.001	5.04 [1.69, 8.39]	0.005	4.65 [1.91, 7.39]	0.002
Serum vitamin B12 (pg/mL)	0.31 [−1.25, 1.87]	0.69	0.27 [−1.31, 1.86]	0.73	0.20 [−1.41, 1.81]	0.804	−0.14 [−1.72, 1.43]	0.851

Model 1: Crude model; Model 2: Adjusted for age, sex; Model 3: Adjusted for model 2 plus race, education, smoking status, drinking status, family income and physical activity; Model 4: Adjusted for model 2 plus TC, ALT, chronic disease status (diabetes, hypertension, CKD, obesity), energy (kcal/d) and dietary vitamins intake.

### Logistic regression analyses

Table 3 presents the results from logistic regression analyses examining the correlation between dietary intake of MedHi food and clinically relevant low serum vitamin groups. When MedHi consumption was analyzed as a continuous variable, each one-unit increase in natural log-transformed MedHi consumption was associated with a 3% (95% CI: 1–5%), 3% (95% CI: 2–5%), and 4% (95% CI: 2–6%) lower risk of being in the low serum vitamin A, D, and E groups, respectively, after multivariate adjustment. No significant association was observed with the low serum vitamin B12 group. Furthermore, when MedHi consumption was categorized into three groups based on the quantity of MedHi food intake, the risk of having low serum FSVs levels (A, D, E) was significantly lower in the G3 highest consumption group compared with the G1 non-consumption group across all four models, as detailed in Table 4. Furthermore, when categorized by the general microbial quantity of their diets, the risk of having low serum FSVs groups (A, D, E) was significantly lower in the moderately higher consumption groups compared to the lower consumption group.

**Table 3:** Regression analysis showing the association between dietary intake of live microbes with lower levels of serum FSVs.

Variables	Per one-unit increment in natural log-transformed MedHi(g)							
	Model 1	*p*	Model 2	*p*	Model 3	*p*	Model 4	*p*
Low serum vitamin A group	0.96 [0.94, 0.97]	<0.001	0.96 [0.94, 0.97]	<0.001	0.97 [0.95, 0.99]	0.003	0.97 [0.95, 0.99]	0.003
Serum vitamin D deficiency	0.93 [0.92, 0.94]	<0.001	0.93 [0.92, 0.95]	<0.001	0.96 [0.94, 0.97]	<0.001	0.97 [0.95, 0.98]	<0.001
Low serum vitamin E group	0.93 [0.92, 0.95]	<0.001	0.94 [0.92, 0.96]	<0.001	0.96 [0.94, 0.98]	0.002	0.96 [0.94, 0.98]	0.002
Low serum vitamin B12 group	1.00 [0.98, 1.02]	0.792	1.00 [0.98, 1.02]	0.797	1.00 [0.98, 1.02]	0.915	1.00 [0.98, 1.02]	0.832

Model 1: crude model; Model 2: Adjusted for age, sex; Model 3: Adjusted for model 2 plus race, education, smoking status, drinking status, family income and physical activity; Model 4: Adjusted for model 2 plus TC, ALT, chronic disease status (diabetes, hypertension, CKD, obesity), energy (kcal/d) and dietary vitamins intake.

**Table 4:** Regression analysis showing the association between dietary intake of live microbes with lower levels of serum FSVs.

Variables	Model 1	*p*	Model 2	*p*	Model 3	*p*	Model 4	*p*
Lower serum vitamin A group								
Categories								
G1	1		1		1		1	
G2	0.93 [0.75, 1.14]	0.47	0.94 [0.76, 1.16]	0.552	1.08 [0.87, 1.33]	0.464	1.10 [0.85, 1.42]	0.438
G3	0.60 [0.53, 0.68]	<0.001	0.64 [0.56, 0.73]	<0.001	0.81 [0.69, 0.94]	0.007	0.78 [0.65, 0.92]	0.007
*p* for trend	<0.001		<0.001		0.004		0.005	
Dietary intake of live microbes group								
Low	1		1		1		1	
Moderate	0.79 [0.69, 0.90]	0.001	0.80 [0.70, 0.92]	0.003	0.87 [0.76, 1.01]	0.068	0.86 [0.73, 1.01]	0.069
High	0.70 [0.61, 0.81]	<0.001	0.65 [0.56, 0.76]	<0.001	0.76 [0.65, 0.90]	0.002	0.76 [0.64, 0.90]	0.003
*p* for trend	<0.001		<0.001		0.002		0.002	
Serum vitamin D deficiency								
Categories								
G1	1		1		1		1	
G2	0.75 [0.63, 0.88]	0.001	0.73 [0.62, 0.86]	<0.001	0.79 [0.67, 0.94]	0.009	0.79 [0.66, 0.94]	0.01
G3	0.60 [0.55, 0.66]	<0.001	0.63 [0.57, 0.69]	<0.001	0.76 [0.68, 0.85]	<0.001	0.80 [0.72, 0.89]	<0.001
*p* for trend	<0.001		<0.001		<0.001		<0.001	
Dietary intake of live microbes group								
Low	1		1		1		1	
Moderate	0.68 [0.61, 0.76]	<0.001	0.71 [0.64, 0.79]	<0.001	0.78 [0.70, 0.87]	<0.001	0.81 [0.72, 0.90]	<0.001
High	0.55 [0.49, 0.62]	<0.001	0.56 [0.50, 0.63]	<0.001	0.74 [0.65, 0.85]	<0.001	0.79 [0.69, 0.90]	0.001
*p* for trend	<0.001		<0.001		<0.001		<0.001	
Lower serum vitamin E group								
Categories								
G1	1		1		1		1	
G2	0.93 [0.75, 1.14]	0.47	0.94 [0.76, 1.16]	0.552	1.08 [0.87, 1.33]	0.464	1.10 [0.85, 1.42]	0.438
G3	0.60 [0.53, 0.68]	<0.001	0.64 [0.56, 0.73]	<0.001	0.81 [0.69, 0.94]	0.007	0.78 [0.65, 0.92]	0.007
*p* for trend	<0.001		<0.001		<0.001		<0.001	
Dietary intake of live microbes group								
Low	1		1		1		1	
Moderate	0.65 [0.57, 0.75]	<0.001	0.72 [0.62, 0.83]	<0.001	0.81 [0.69, 0.94]	0.008	0.79 [0.66, 0.96]	0.018
High	0.68 [0.58, 0.80]	<0.001	0.65 [0.55, 0.77]	<0.001	0.80 [0.67, 0.95]	0.012	0.80 [0.66, 0.96]	0.022
*p* for trend	<0.001		<0.001		0.006		0.013	

Model 1: crude model; Model 2: Adjusted for age, sex; Model 3: Adjusted for model 2 plus race, education, smoking status, drinking status, family income and physical activity; Model 4: (for serum vitamins): Adjusted for model 3 plus TC, ALT, chronic disease status (diabetes, hypertension, CKD, obesity), energy (kcal/d) and dietary vitamins intake.

**Figure 3:**
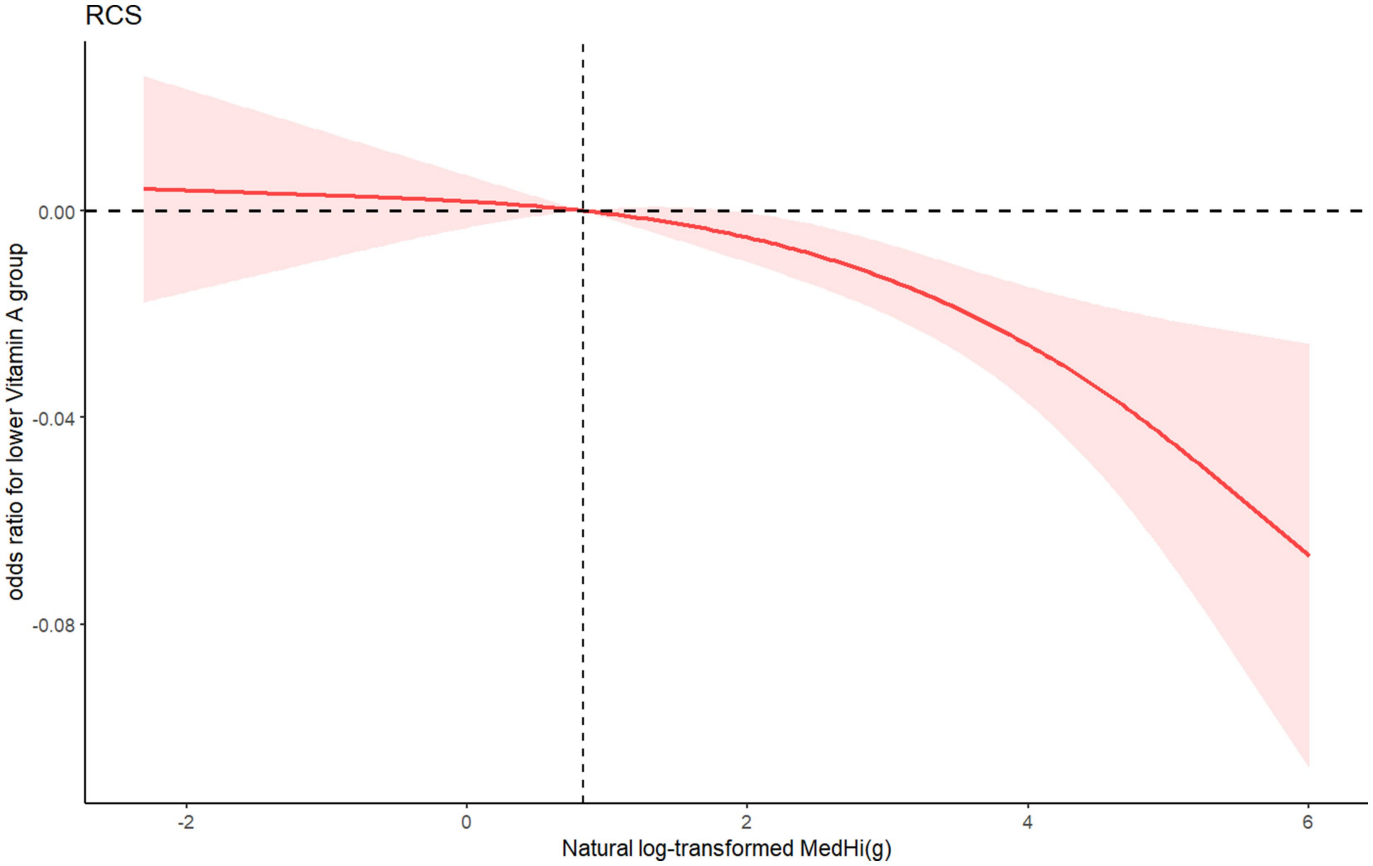
RCS showing the association between natural log-transformed MedHi food intake and odds ratio of lower serum vitamin A group.

**Figure 4:**
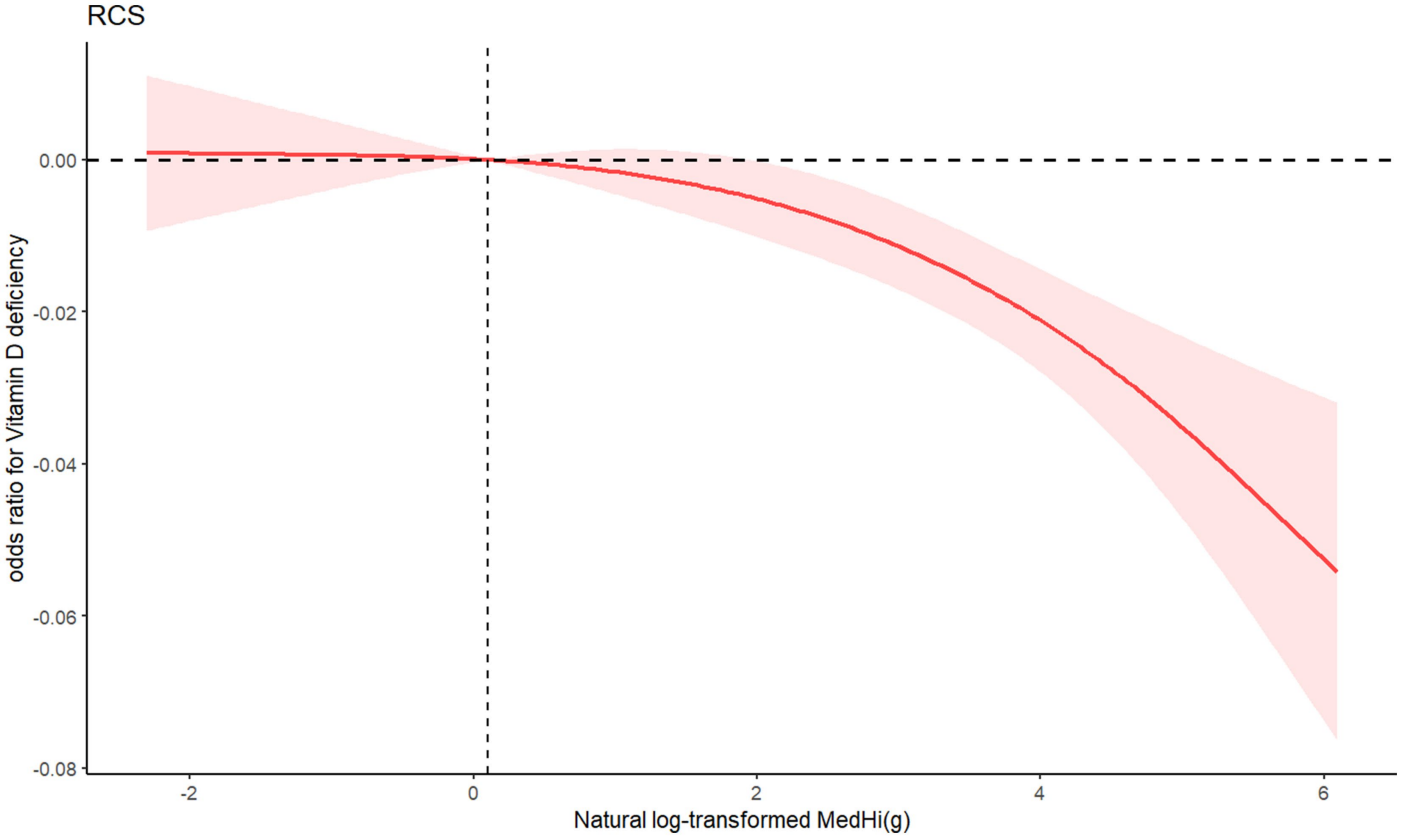
RCS showing the association between natural log-transformed MedHi food intake and odds ratio of vitamin D deficency.

**Figure 5:**
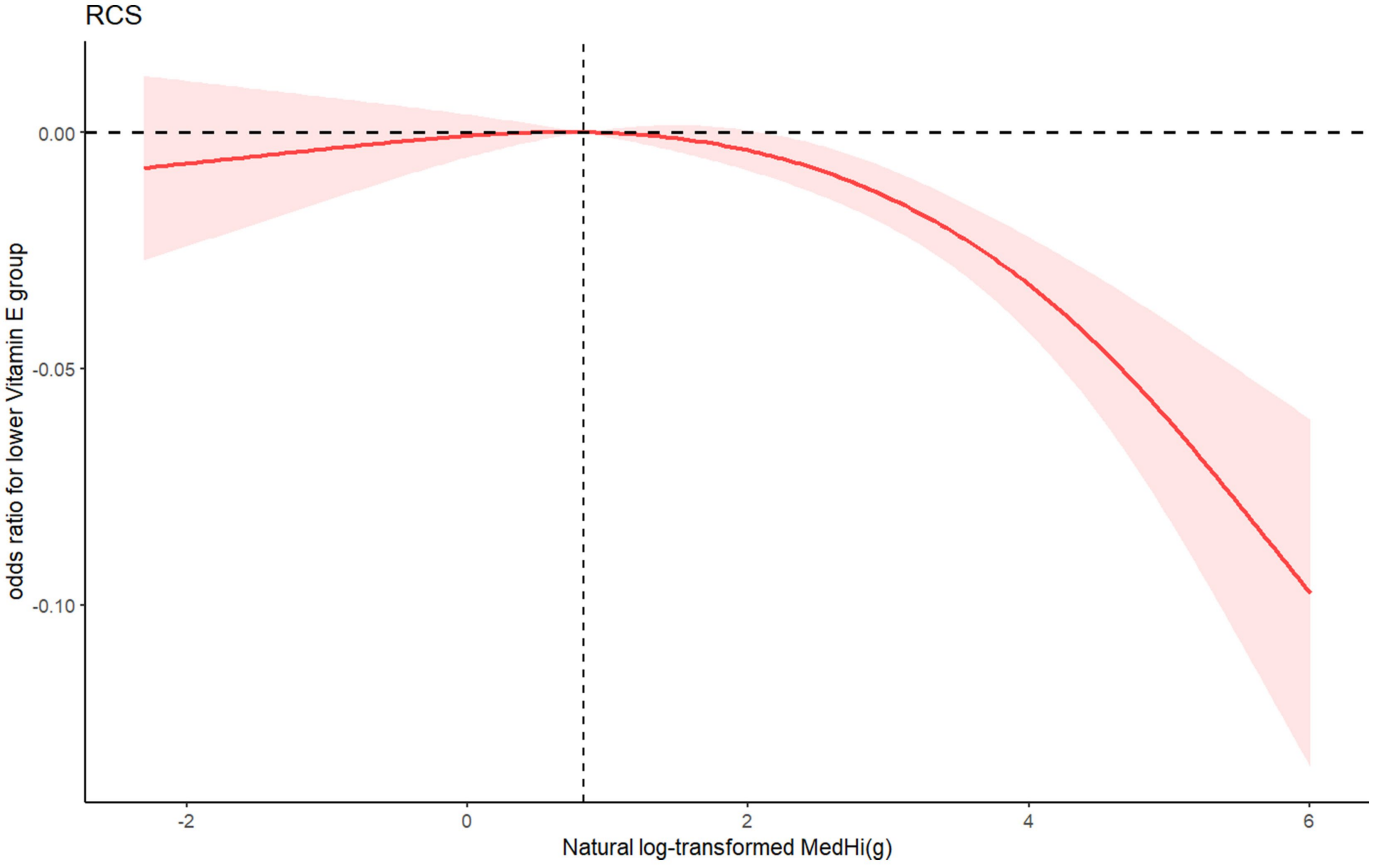
RCS showing the association between natural log-transformed MedHi food intake and odds ratio oflower serum vitamin E group.

Restricted cubic splines (RCSs) demonstrated the relationship between dietary intake of MedHi food and serum levels of FSVs (A, D, E). The exposure-response curves for MedHi consumption showed a consistent decreasing trend in the prevalence of low serum levels of these FSVs, as illustrated in Figures 3–5.

### Sensitivity analyses

Given the strong correlation between time spent outdoors for physical activity and serum vitamin D levels (22), it is however notable that the NHANES database has collected physical activity data which did not specify as outdoor physical activity. In the sensitivity analysis, the association between the dietary intake of live microbes and serum vitamin D levels was examined with time spent outdoors included as a covariate in the statistical analysis (Supplementary Table S3). However, additional adjustments for time spent outdoors did not significantly alter the outcomes.

## Discussion

In this nationally representative cross-sectional study, we observed that dietary intake of live microbes was associated with higher serum levels of FSVs (A, D, and E), while no significant association was found with vitamin B12, a water-soluble vitamin, suggesting that the observed associations may be specific to FSVs. These findings suggested that live microbe consumption may specifically affect the status of FSVs. To our knowledge, this is the first population-based study in the United States to explore these associations, providing novel insights into the relationship between the dietary intake of live microbes and FSVs.

Several mechanisms may underlie these associations. Absorption depends heavily on intestinal function and gut microbiota activity (8, 23). The gut microbiota can influence bile acid metabolism, lipid digestion, mucosal integrity, and transporter expression, all of which modulate the absorption and bioavailability of FSVs (7, 24, 25). Moreover, certain live dietary microbes may directly interact with the gut environment, enhancing vitamin uptake or modifying gut permeability, thereby facilitating vitamin absorption. Although vitamin D has been shown to regulate microbiota composition, and higher vitamin D levels are associated with increased gut microbial diversity (26), few studies have specifically evaluated the role of the dietary intake of live microbes on FSVs, particularly in large-scale epidemiologic settings.

Our findings are consistent with previous mechanistic and observational studies suggesting bidirectional interactions between the gut microbiota and host vitamin metabolism (7, 8, 26, 27). However, our results extend prior work by demonstrating that the dietary intake of live microbes may be a modifiable dietary factor associated with improved FSVs status.

This study has several strengths, including the large, nationally representative NHANES sample, standardized biomarker assessments, and comprehensive adjustment for demographic, lifestyle, laboratory, and dietary covariates. Additionally, we examined both fat- and water-soluble vitamins to better evaluate the specificity of the observed associations.

However, several limitations must be acknowledged. First, due to the cross-sectional nature of NHANES, causality cannot be established. Second, the assessment of the dietary intake of live microbes relied on a food frequency approach and estimates from Sanders’ classification, which may not capture all sources or strains of live microbes. Third, the FSVs were measured across different NHANES cycles, requiring separate analytic groups, and preventing combined multivariate modeling across vitamins. Finally, residual confounding by unmeasured factors cannot be entirely excluded.

In summary, our findings suggest that the dietary intake of live microbes was associated with higher serum levels of FSVs in the U. S. population. These results highlight a potentially modifiable nutritional target for improving micronutrient status. Prospective longitudinal studies and interventional trials are warranted to further investigate the causal relationships and underlying mechanisms involved.

## Conclusion

In conclusion, this study supports an association between the dietary intake of live microbes and increased serum levels of FSVs. Specifically, each one-unit increase in the natural log-transformed MedHi food intake was associated with increases of 0.17 μg/dL in vitamin A, 0.36 nmol/L in vitamin D, and 4.65 μg/dL in vitamin E. These associations may contribute to reducing deficiencies in these vitamins. Prospective long-term studies are warranted to further investigate the efficacy of dietary live microbe intake in improving serum FSVs levels.

## Data Availability

The datasets presented in this study can be found in online repositories. The names of the repository/repositories and accession number(s) can be found in the article/Supplementary material.

## References

[1] Girgis CM Clifton-Bligh RJ Hamrick MW Holick MF Gunton JE. The roles of vitamin D in skeletal muscle: form, function, and metabolism. Endocr Rev. (2013) 34:33–83. doi: 10.1210/er.2012-1012, PMID: 23169676

[2] Carazo A Macáková K Matoušová K Krčmová LK Protti M Mladěnka P. Vitamin a update: forms, sources, kinetics, detection, function, deficiency, therapeutic use and toxicity. Nutrients. (2021) 13:1703. doi: 10.3390/nu13051703, PMID: 34069881 PMC8157347

[3] Ismailova A White JH. Vitamin D, infections and immunity. Rev Endocr Metab Disord. (2022) 23:265–77. doi: 10.1007/s11154-021-09679-5, PMID: 34322844 PMC8318777

[4] Miyazawa T Burdeos GC Itaya M Nakagawa K Miyazawa T. Vitamin E: regulatory redox interactions. IUBMB Life. (2019) 71:430–41. doi: 10.1002/iub.2008, PMID: 30681767

[5] Eshak ES Iso H Muraki I Tamakoshi A. Fat-soluble vitamins from diet in relation to risk of type 2 diabetes mellitus in Japanese population. Br J Nutr. (2019) 121:647–53. doi: 10.1017/S000711451800377X, PMID: 30567614

[6] Holick MF Chen TC. Vitamin D deficiency: a worldwide problem with health consequences. Am J Clin Nutr. (2008) 87:1080s–6s. doi: 10.1093/ajcn/87.4.1080S, PMID: 18400738

[7] Boughanem H Ruiz-Limón P Pilo J Lisbona-Montañez JM Tinahones FJ Moreno Indias I. Linking serum vitamin D levels with gut microbiota after 1-year lifestyle intervention with Mediterranean diet in patients with obesity and metabolic syndrome: a nested cross-sectional and prospective study. Gut Microbes. (2023) 15:2249150. doi: 10.1080/19490976.2023.2249150, PMID: 37647262 PMC10469434

[8] Stacchiotti V Rezzi S Eggersdorfer M Galli F. Metabolic and functional interplay between gut microbiota and fat-soluble vitamins. Crit Rev Food Sci Nutr. (2021) 61:3211–32. doi: 10.1080/10408398.2020.1793728, PMID: 32715724

[9] Leeuwendaal NK Stanton C O’Toole PW Beresford TP. Fermented foods, health and the gut microbiome. Nutrients. (2022) 14:1527. doi: 10.3390/nu14071527, PMID: 35406140 PMC9003261

[10] Ley RE Lozupone CA Hamady M Knight R Gordon JI. Worlds within worlds: evolution of the vertebrate gut microbiota. Nat Rev Microbiol. (2008) 6:776–88. doi: 10.1038/nrmicro1978, PMID: 18794915 PMC2664199

[11] David LA Maurice CF Carmody RN Gootenberg DB Button JE Wolfe BE. Diet rapidly and reproducibly alters the human gut microbiome. Nature. (2014) 505:559–63. doi: 10.1038/nature12820, PMID: 24336217 PMC3957428

[12] Song X Sun X Oh SF Wu M Zhang Y Zheng W. Microbial bile acid metabolites modulate gut RORγ(+) regulatory T cell homeostasis. Nature. (2020) 577:410–5. doi: 10.1038/s41586-019-1865-0, PMID: 31875848 PMC7274525

[13] Guzior DV Okros M Shivel M Armwald B Bridges C Fu Y. Bile salt hydrolase acyltransferase activity expands bile acid diversity. Nature. (2024) 626:852–8. doi: 10.1038/s41586-024-07017-8, PMID: 38326608

[14] Bellerba F Muzio V Gnagnarella P Facciotti F Chiocca S Bossi P. The association between vitamin D and gut microbiota: a systematic review of human studies. Nutrients. (2021) 13:378. doi: 10.3390/nu13103378, PMID: 34684379 PMC8540279

[15] Mandal S Godfrey KM McDonald D Treuren WV Bjørnholt JV Midtvedt T. Fat and vitamin intakes during pregnancy have stronger relations with a pro-inflammatory maternal microbiota than does carbohydrate intake. Microbiome. (2016) 4:55. doi: 10.1186/s40168-016-0200-3, PMID: 27756413 PMC5070355

[16] Singh P Rawat A Saadaoui M Elhag D Tomei S Elanbari M. Tipping the balance: vitamin D inadequacy in children impacts the major gut bacterial Phyla. Biomedicines. (2022) 10:278. doi: 10.3390/biomedicines10020278, PMID: 35203487 PMC8869474

[17] Pfeiffer CM Lacher DA Schleicher RL Johnson CL Yetley EA. Challenges and lessons learned in generating and interpreting NHANES nutritional biomarker data. Adv Nutr. (2017) 8:290–307. doi: 10.3945/an.116.014076, PMID: 28298273 PMC5347107

[18] Holick MF. Vitamin D deficiency. N Engl J Med. (2007) 357:266–81. doi: 10.1056/NEJMra070553, PMID: 17634462

[19] Holick MF. High prevalence of vitamin D inadequacy and implications for health. Mayo Clin Proc. (2006) 81:353–73. doi: 10.4065/81.3.353, PMID: 16529140

[20] Bischoff-Ferrari HA Giovannucci E Willett WC Dietrich T Dawson-Hughes B. Estimation of optimal serum concentrations of 25-hydroxyvitamin D for multiple health outcomes. Am J Clin Nutr. (2006) 84:18–28. doi: 10.1093/ajcn/84.1.18, PMID: 16825677

[21] Glassock RJ Warnock DG Delanaye P. The global burden of chronic kidney disease: estimates, variability and pitfalls. Nat Rev Nephrol. (2017) 13:104–14. doi: 10.1038/nrneph.2016.163, PMID: 27941934

[22] Ouyang S Li Q Liu Z Yin Y. The relationship between physical activity levels and serum vitamin D levels varies among children and adolescents in different age groups. Front Nutr. (2024) 11:1435396. doi: 10.3389/fnut.2024.1435396, PMID: 39279903 PMC11392726

[23] Reboul E. Proteins involved in fat-soluble vitamin and carotenoid transport across the intestinal cells: new insights from the past decade. Prog Lipid Res. (2023) 89:101208. doi: 10.1016/j.plipres.2022.101208, PMID: 36493998

[24] Zhang T Sun P Geng Q Fan H Gong Y Hu Y. Disrupted spermatogenesis in a metabolic syndrome model: the role of vitamin a metabolism in the gut-testis axis. Gut. (2022) 71:78–87. doi: 10.1136/gutjnl-2020-323347, PMID: 33504491 PMC8666830

[25] Li A Li F Song W Lei ZL Sha QQ Liu SY. Gut microbiota-bile acid-vitamin D axis plays an important role in determining oocyte quality and embryonic development. Clin Transl Med. (2023) 13:e1236. doi: 10.1002/ctm2.1236, PMID: 37846137 PMC10580005

[26] Bora SA Kennett MJ Smith PB Patterson AD Cantorna MT. The gut microbiota regulates endocrine vitamin D metabolism through fibroblast growth factor 23. Front Immunol. (2018) 9:408. doi: 10.3389/fimmu.2018.00408, PMID: 29599772 PMC5863497

[27] Mukherjee A Breselge S Dimidi E Marco ML Cotter PD. Fermented foods and gastrointestinal health: underlying mechanisms. Nat Rev Gastroenterol Hepatol. (2024) 21:248–66. doi: 10.1038/s41575-023-00869-x, PMID: 38081933

