# Supplementary material for: The association between the serum fat-soluble vitamins (A, D and E) and the intake of live microbes: a national population based cross-sectional study
Source: Front Nutr. 2025 Jul 29;12:1593461. doi: 10.3389/fnut.2025.1593461 (PMC12342190; doi:10.3389/fnut.2025.1593461)
Supplement: Supplementary file 1 [file Table_1.docx]

Table S1 Demographic characteristics of Group 2 (NHANES 2009–2018)

| **Variables** | **Total** | MedHi  **groups** | | | ***P*** |
| --- | --- | --- | --- | --- | --- |
|  |  | **G1** | **G2** | **G3** |  |
| Age (years) | 46.69(0.31) | 44.90(0.35) | 48.26(0.41) | 46.84(0.42) | < 0.0001 |
| Sex |  |  |  |  | < 0.0001 |
| Male | 9558(50.09) | 4093(55.61) | 3594(48.07) | 1871(45.60) |  |
| Female | 9442(49.91) | 3423(44.39) | 3749(51.93) | 2270(54.40) |  |
| Race |  |  |  |  | < 0.0001 |
| Non-Hispanic White | 7975(66.71) | 2843(61.46) | 2987(65.42) | 2145(75.54) |  |
| Other Race | 11025(33.29) | 4673(38.54) | 4356(34.58) | 1996(24.46) |  |
| Education |  |  |  |  | < 0.0001 |
| Less than high school | 1619( 4.28) | 698(5.36) | 746(5.00) | 175(1.81) |  |
| High school or equivalent | 7223(34.72) | 3329(42.51) | 2661(32.78) | 1233(27.07) |  |
| College or above | 10158(61.00) | 3489(52.13) | 3936(62.21) | 2733(71.12) |  |
| Family income-to-poverty ratio |  |  |  |  | < 0.0001 |
| <1.3 | 6249(23.31) | 2962(30.12) | 2258(21.42) | 1029(16.90) |  |
| 1.3-3.5 | 7170(35.59) | 2886(38.10) | 2800(35.52) | 1484(32.33) |  |
| >3.5 | 5581(41.10) | 1668(31.78) | 2285(43.06) | 1628(50.77) |  |
| Smoking status |  |  |  |  | < 0.0001 |
| Never | 10694(56.06) | 3956(51.23) | 4265(57.91) | 2473(59.88) |  |
| Former | 4417(24.11) | 1609(22.19) | 1827(26.04) | 981(23.94) |  |
| Current | 3889(19.83) | 1951(26.59) | 1251(16.05) | 687(16.18) |  |
| Drinking status |  |  |  |  | < 0.0001 |
| Non-alcohol intake | 2725(10.76) | 1150(11.80) | 1077(11.18) | 498( 8.78) |  |
| Former alcohol intake | 2733(11.82) | 1216(13.31) | 1030(12.09) | 487( 9.45) |  |
| Mild alcohol intake | 6576(37.04) | 2300(32.18) | 2640(38.27) | 1636(41.77) |  |
| Heavy alcohol intake | 6966(40.39) | 2850(42.71) | 2596(38.47) | 1520(40.01) |  |
| Recreational activity |  |  |  |  | < 0.0001 |
| No | 9727(45.88) | 4308(53.68) | 3628(43.75) | 1791(38.51) |  |
| Moderate | 4915(27.75) | 1723(24.49) | 1993(28.91) | 1199(30.46) |  |
| Vigorous | 4358(26.37) | 1485(21.83) | 1722(27.34) | 1151(31.03) |  |
| Obesity |  |  |  |  | < 0.0001 |
| No | 11373(60.28) | 4337(57.10) | 4455(61.83) | 2581(63.71) |  |
| Yes | 7471(39.10) | 3104(42.90) | 2829(38.17) | 1538(36.29) |  |
| CKD |  |  |  |  | 0.05 |
| No | 15509(84.91) | 6058(85.57) | 5969(85.37) | 3482(87.53) |  |
| Yes | 3226(13.80) | 1343(14.43) | 1270(14.63) | 613(12.47) |  |
| Diabetes |  |  |  |  | < 0.001 |
| No | 15412(85.31) | 6091(85.21) | 5847(83.88) | 3474(87.46) |  |
| Yes | 3588(14.69) | 1425(14.79) | 1496(16.12) | 667(12.54) |  |
| Hypertension |  |  |  |  | 0.01 |
| No | 11248(62.97) | 4367(61.83) | 4314(62.17) | 2567(65.70) |  |
| Yes | 7746(36.99) | 3148(38.17) | 3024(37.83) | 1574(34.30) |  |
| outdoorh_group |  |  |  |  | 0.03 |
| xy0.5 | 1502( 7.12) | 644(11.26) | 573( 9.80) | 285( 8.29) |  |
| 0.5~1 | 539( 2.88) | 222(3.73) | 195(4.41) | 122(3.89) |  |
| 1~2 | 1659( 9.74) | 604(12.17) | 622(13.71) | 433(15.39) |  |
| 2~4 | 3329(19.85) | 1292(26.67) | 1248(27.55) | 789(29.31) |  |
| 4~6 | 2050(12.69) | 792(17.40) | 782(18.12) | 476(17.57) |  |
| 6~8 | 1244( 7.49) | 508(10.73) | 435( 9.51) | 301(11.32) |  |
| 8~10 | 903( 5.76) | 352(8.33) | 369(8.77) | 182(6.61) |  |
| 10~12 | 575( 3.30) | 256(5.14) | 214(4.59) | 105(3.89) |  |
| 12~14 | 376( 2.13) | 178(3.24) | 135(2.72) | 63(2.96) |  |
| 14~16 | 123( 0.71) | 62(1.34) | 39(0.81) | 22(0.76) |  |
| TC (mmol/L) | 4.98(0.02) | 4.90(0.02) | 5.00(0.02) | 5.05(0.03) | < 0.0001 |
| ALT (U/L) | 25.50(0.21) | 26.16(0.37) | 25.31(0.24) | 24.91(0.53) | 0.11 |
| MedHi (g/d) | 94.25(1.73) | 0.00(0.00) | 133.36(2.26) | 164.58(2.72) | < 0.0001 |
| Vitamin D intake (mcg) | 4.55(0.07) | 4.22(0.10) | 4.67(0.11) | 4.82(0.13) | < 0.001 |
| Serum vitamin D (nmol/L) | 63.93(0.60) | 59.73(0.76) | 65.54(0.71) | 67.26(0.61) | < 0.0001 |
| Energy intake (kcal/d) | 2147.25(8.57) | 2105.23(15.31) | 2133.00(14.66) | 2223.49(17.77) | < 0.0001 |
